# Lectures

**Published:** 1844-06-29

**Authors:** Benjamin Collins Brodie

**Affiliations:** Consulting-Surgeon of the Hospital


### CLINICAL LECTURES AND REPORTS.

### LECTURES

*Delivered in the Theatre of St. George's Hospital, in the session* 1843-44,

BY SIR BENJAMIN COLLINS BRODIE,

Consulting-Surgeon of the Hospital.

*Non-malignant diseases of the tongue. Paralysis and its causes. Paraplegia, its origin and progress. Softening of the spinal nervous substance. Tabes dorsalis. Cases of paraplegia. Paralysis from carcinomatous vertebræ. Case of general paralysis. Lumbago a precursor of paralysis. Paraplegia with hysteria and chlorosis.*

Gentlemen,—In my last lecture I spoke of diseases of the tongue. I should have mentioned that other kinds of tumours than those I there described occur in that organ, just as they do in other parts of the body. Their formation in the tongue is not a frequent occurrence; nevertheless, you meet with them sometimes. A gentleman came to me with a tumour of the tongue, which was distinguished from common scirrhus by its being further from the surface, and very distinctly circumscribed: still, from the hardness of the tumour, I was led to suspect that it might be of a malignant nature. Had I found the same kind of tumour in the female breast I should have said that it was scirrhus; but as it had not the character of common scirrhus of the tongue I entertained doubts upon the subject. As an experiment I gave the patient tincture of iodine, eight or ten drops three times a day, gradually increasing the dose to twenty drops. After taking this for a short time the tumour appeared reduced in size; and on continuing the medicine for some time longer it was still more reduced, and it ultimately disappeared entirely. What the nature of the tumour was I do not pretend to say. I may mention one circumstance connected with this case, by way of putting you on your guard as to the use of tincture of iodine. The patient wished to go into the country, to which I gave permission, provided he would have a medical attendant to look after him while taking this remedy, adding that I could not sanction any patient of mine taking this medicine except under medical observation. He took the iodine without placing himself under medical care; and its action not being properly watched, he one day had a paralytic stroke. He instantly left off the medicine, and he ultimately recovered. This is only one case of many which I might mention, to show that iodine often produces powerful effects on the nervous system, and that it is not to be taken—at least in large doses—without considerable caution. I remember seeing a patient who had a large elastic tumour, or some fluctuation in the tongue, of considerable size, apparently as big as a nutmeg. It was perceptible chiefly on the lower surface of the organ. The surgeon, under whose care the patient was, divided the tongue over the tumour to see what it was, and out came a cyst containing fluid—I suppose an hydatid. The patient got well. These observations are intended to finish the subject to which I before called your attention.

#### PARALYSIS.

I now enter upon another topic. When such a change takes place in the nervous system that the mandates of the will are not conveyed to the muscles, we say that there is paralysis. Paralysis may be, and generally is, attended with a loss of sensation also to a greater or less extent; but this is not a matter of course. The nerves of sensation may be affected without involving the nerves of motion, and *vice versâ*.

Paralytic affections may depend, as you may suppose, on various causes. Mere general deficiency of nervous agency; the accidental division of a nerve of the spinal cord; pressure upon any part of the nervous system; tumours or other morbid alterations of structure in the brain and spinal marrow, will produce paralysis.

Where there is a tumour or morbid alteration of structure, in some instances, the paralysis will come on gradually; but it is a remarkable circumstance that in many instances that is not the case. Disease is going on, perhaps, for months, or even years, and all at once there is a sudden stroke of paralysis. For example, the late Dr. Wollaston, the eminent philosopher, had a disease of the brain, which proved to be a tumour situated in one optic thalamus, and it produced in him a remarkable effect. He saw one-half of an object, and not the other half. He used frequently during life to talk to me on the subject of this peculiarity of vision. He had it when a boy at school, but when sixty years of age he was all at once seized with paralysis in one arm, that extended, and he died. On the post-mortem examination we found a tumour as large as a walnut connected with one optic thalamus. A gentleman consulted me last year who had, all at once, become paralytic in the lower limbs. I need not detail the case; he ultimately died, and on examining the body I found a tumour in the middle of the spinal cord, at the back, which evidently must have been growing for years. This was proved by other symptoms, but there had been no paralysis. I attended a gentleman for diseased prostate gland; he was in a very miserable hypochondriacal condition, and used to cry without any evident reason for it. One day on going to the close-stool he all at once became paralytic on one side of the body, and he died. On examination we found *ramollissement* of one complete hemisphere of the cerebrum.

The sudden occurrence of paralysis in these cases is to be accounted for in the following manner:—The tumour or morbid alteration of structure goes on in the brain, and then there is a sudden effusion of serum into the ventricles. In Dr. Wollaston's case the tumour grew so gradually that it did not affect the functions of the brain; but all at once it projected into the ventricles so as to produce irritation of the lining membrane, and then there was a sudden effusion of water into the ventricles. It was the same with the gentleman who died from ramollissement of the brain. That must have been going on for months, and no doubt produced low spirits, a disposition to weep, &c. On examining the body after death we found the ventricles distended with water, and I conclude that it was the sudden effusion of water there that caused the sudden paralysis. I know of some other cases in which water has been effused into the ventricles of the brain, independently of inflammation, in a very short space of time. The ancient writers distinguished between sanguineous and serous apoplexy. In the former, blood is extravasated from the rupture of a vessel in the brain; in the latter, water is effused into the ventricles, and both occurrences may take place suddenly. I have known a person become quite apoplectic in a few hours, having been perfectly well before; and on examining the body after death, I have found the ventricles distended with serum.

Again, the sudden occurrence of paralysis in the case where there was a tubercle in the spinal cord, I apprehend, was to be explained by this circumstance, that all below the part where the tubercle was situated was in a state of softening, or *ramollissement*, as the French call it; but I shall have to advert to this subject again presently.

Different names have been given to different forms of paralysis. You hear of *hemiplegia*—half the body being struck. Sometimes there is paralysis in one leg, one arm, or down one side of the body, and not the other side, and this form of paralysis is generally called *hemiplegia*. It always depends on disease in the brain itself. The right side of the brain belongs to the left side of the body, and *vice versâ*. If the left leg and the left arm, therefore, become paralytic, you conclude, as a matter of course, that the disease is on the right side of the brain. Another form of paralysis is called *paraplegia*. That word has been used rather indefinitely, but still I believe that every one who has employed it has meant to say that the paralysis was not confined to one side of the body, but exists on both sides. The Greek preposition παρα signifies "stroke across."

Now, it is to the various cases that are confounded with one another under the name of paraplegia to which I wish to call your attention in this and, probably, in my next lecture.

You will often find a person with these symptoms,—I think I see such a case every month of my life. The patient complains of a difficulty of walking; he finds that he stumbles easily. When he attempts to use his limbs he sometimes finds that he cannot carry his intention into effect; the muscles do not exactly obey his will. He finds that he does not stand steady; that he must put his feet asunder in order that they may be wide, otherwise the centre of gravity is apt to go too much on one side. This difficulty increases, at last he walks very unsteadily indeed; the muscles of the lower limbs become flaccid; the weakness of the muscles extends upwards, and generally there is a loss of sensation. For a long time the latter is not complete, nor is there a complete loss of the power of motion, but the disease is gradually creeping on. By and by the patient complains of a loss of power below his waist, and not only has he a difficulty in walking, but there is a difficulty in making water; he cannot command the bladder, the urine runs away involuntarily, wets his clothes, wets the bedclothes, and makes him offensive to himself and to others. This generally happens from the bladder being overloaded, and not being capable of emptying itself; though sometimes it is the reverse; the bladder is actually empty, and continues so, for the urine runs through without distending it. Generally, however, it is an overloaded bladder that produces incontinence of urine. The patient then has a sense of constriction as if a hoop were bound round his waist. That is a very constant symptom in these cases. Then he will complain of a sensation as though a ligature were bound round each thigh and each leg, and there is increasing numbness, with a sense of weight in the feet.

In some instances the disease remains just as I have described it; and I have known persons go on in this way for many years. I remember a gentleman who had just the symptoms I have mentioned, respecting whom I was consulted, but for whom no good could be done, and I used to see him crawling about the streets for years afterwards. But in other cases the disease goes on; the lower limbs become completely paralytic; then the upper limbs become affected; first one arm and then the other. In some of these cases the bowels are exceedingly costive; they are not to be acted upon, even by the strongest medicine, and very frequently there are pains in the abdomen. Sometimes you find the disease making rapid and at other times slow progress.

Thus I have given you a general description of the symptoms, such as are applicable to the majority of cases of paraplegia with which you will meet, commencing in the lower limbs. We now come to consider what are the different causes on which these symptoms may depend, and what the different diseases that are indicated in this manner.

One, and I believe the most common cause, is that I have mentioned—a morbid change of the minute structure of the spinal cord; that is to say, softening, or *ramollissement*. The change that occurs at other times in the brain takes place in the spinal cord after a concussion of the spine. A very common effect of concussion is to injure its minute structure, and then to a greater or less extent it dissolves into a substance like cream. In this state of softening it first loses its natural consistency, but still retains the character of solid substance. By and by it becomes completely melted down to a substance like cream; the membranes can hardly be lifted out, and when placed in water the spinal cord floats, and the membranes remain by themselves. What produces this softening I cannot say. Some have said that it is inflammation, but certainly there are no marks of inflammation; there is no unusual vascularity preceding or accompanying the softening; there are no vessels loaded with blood, and, indeed, the parts are rather less vascular than natural. All that can be said is, there is some peculiar change of structure, the proximate cause of which we cannot explain, nor very often the remote cause. A young lady had this state of the spinal cord, and ultimately died from it. She was a healthy young woman in other respects, and there was nothing to explain it. There is one very common cause of it—not in young women but in men—men who rank among what is called the *better* classes, which, I suppose, means only that they are richer than others; at any rate, they are not better in the point I am going to mention. There is a class of people, in London especially, who have no employment, who have large fortunes, and who spend half their time in intriguing with women; and in many instances you may trace the disease of the spinal cord to over-indulgence in sexual intercourse. Though we know more of the appearances after death than did the ancients, yet they very well described paralysis arising from this cause when they spoke of it as *tabes dorsalis*.

That is one cause of paraplegic symptoms, but from what other causes may they arise? A gentleman had formerly some pain in the back, or some symptoms which led a surgeon to apply a caustic issue in the neighbourhood of the spine. This was almost forgotten, but about two years ago, in walking, one of his feet gave way, and if his brother had not been with him he would have fallen to the ground; but he was very well again afterwards. By and by, however, he was seized with violent pain around the waist, and it was treated, without any relief, as rheumatic pain. After a time he became completely paralytic in both limbs, he lay in bed for a few days, and then recovered, so that he could walk about the room. This did not last long, he again became paralytic, the bowels were constipated, and no medicine would act upon them. The secretions from the bowels became black, like tar, the urine alkaline, and he died. This was the case which I mentioned just now. On examining the body after death there was a tubercle in the spinal cord, which no doubt had been growing for years. It was a hard solid tubercle, and below it the spinal cord was soft. I presume that the pain which preceded the paralysis indicated the commencement of the softening of the cord below the tubercle. I have seen other cases of medullary tumours around the spinal cord producing paraplegia of the parts below.

Another cause of this affection is an unnatural effusion of fluid into the theca vertebralis. A gentleman was brought to London completely paralytic in the lower limbs; he could not even turn in bed. By and by the upper limbs became paralytic, and he ultimately died. On a post-mortem examination I found no morbid appearances, except an immense secretion of fluid within the theca vertebralis; the dura mater and the arachnoid membrane lining it were also entirely distended with fluid, so that when the posterior part of the ventricle was removed the fluid bulged into the opening. It was not measured, but a large quantity of fluid ran out of the theca vertebralis when the membranes were opened. There was no other disease either of the brain or the spinal marrow, and what produced this unusual quantity of fluid I do not know; there may have been some disease in the minute structure which we could not discover. Sir Astley Cooper informed me of a similar case.

Paraplegia sometimes occurs in patients who labour under carcinoma. A gentleman had a diseased prostate gland; it was much enlarged and indurated, and there was great pain in the region of the prostate. After a time he was seized with severe pains in the back and in the limbs, such as patients frequently have who labour under carcinoma,—intense agonising pain, which nothing will relieve. These pains, in fact, depend on carcinomatous disease in the bone, and the bones of patients thus affected will break from merely turning in bed; I have known this occurrence to take place in the femur. This gentleman, with disease of the prostate, suddenly became paralytic in the lower limbs, and died; but there was no post-mortem examination of the body. A lady whom I attended last year was suffering from a hopeless case of carcinoma in the breast, and agonising carcinomatous pains in the limbs. One day she became paralytic, lost the use of the lower limbs, and died. Here, also, there was no post-mortem examination. But I met with the following case:—A lady consulted me concerning a scirrhous tumour in the breast. She had gone through the operation for it a year or two before; the disease had returned, and therefore, as far as this was concerned, nothing could be done. By and by there were pains in the limbs and in the back. One night, all at once she lost the use of her lower limbs—could not move them. She died; I was engaged at the time, and could not attend the examination of the body, but Mr. Cutler conducted it. He found, as we had expected, carcinoma of the bones of the spine, and the disease had extended to the dura mater. The carcinomatous bones did not press on the spinal cord; but the disease had produced irritation of the arachnoid membrane, and there was a large secretion of bloody fluid into the theca vertebralis near the cavity of the arachnoid. It was evident that the collection of fluid in the theca vertebralis had been the cause of the paraplegia.

It has been said that paraplegia—paralysis of the lower limbs generally—depends on disease of the brain and not of the spinal marrow. This was maintained by Dr. Baillie, and published in a paper of his in the Transactions of the College of Physicians; but he gives no facts on which the opinion is grounded. It seems to have been a notion taken up by him without any facts to justify it. However, there is reason to believe that, under certain circumstances, disease of the brain may produce paralysis in the lower limbs before it produces it in the upper. I examined the body of a man who was paraplegic, and I found water in the ventricles of the brain, but no disease connected with the spinal marrow. That you may have disease, however, in the brain and in the spinal marrow, combined in the same individual, there can be no doubt. Some of those young men who, from foolish habits, become paraplegic in the lower limbs, have also cerebral symptoms. There may be softening of the lower half of the spinal marrow, and of a good part of the brain. I think that if there is an entire absence of cerebral symptoms you have a right to conclude that the disease does not exist in the brain, but is confined to the parts below; if, however, the patient says he has double vision, if you find one pupil dilated and not the other, and there be pain in the head and giddiness, you have a right to conclude that there is disease in the brain; but still if there were absolute paralysis I should conclude that there was disease in the spine also.

The case which I am about to mention is a very remarkable one. About nine years ago I was sent for into Lincolnshire to see a gentleman who was paralytic in the lower limbs. The symptoms of paralysis had exhibited themselves eight years before, and at the same time there was pain referred to the epigastrium. The disease had now extended upwards, the arms were beginning to be affected, and there was also dilatation of the pupil of one eye; but at the commencement it was a case of regular paraplegia. Neither my advice nor that of any one else did any good, and the disease was left alone. Ten years afterwards his wife was very ill, and he was brought with her to London. She came for medical advice; but his case being considered hopeless he did not consult any one. He was now completely paralytic in his limbs and arms, he could scarcely speak, and he could only just swallow. He lay as though the head were alive and nothing else. His wife died, and he soon followed. I obtained leave to examine the body. Mr. Tatum and another friend accompanied me. We all three made a very careful examination. What we might have found if the spinal cord and brain had been macerated in alcohol, and if we had traced the fibres and examined them with a microscope, I cannot pretend to say; but, with such an examination as we could make in a private house in the course of a couple of hours devoted to it, we could not detect any morbid appearances at all. The spinal cord seemed rather smaller in size than usual, there was some little effusion between the pia mater and the arachnoid, and at the upper part of the spinal cord there was manifestly a blush. The patient had felt for a considerable time pain in the epigastrium, and I thought that might indicate some disease in the plexus there. We took it home with us; Mr. Tatum dissected it with the greatest care, but nothing could be discovered. Do not, however, suppose that I believe this to be a mere functional disease, because we see nothing after death. The minute organisation of the brain and spinal marrow is not visible to the naked eye, and even with the microscope you can only trace it a little way. I doubt not that there was some defect in the minute organisation of the body, some change of structure not perceptible to us. I cannot suppose that such a train of symptoms could occur from mere functional disease.

Another cause of paraplegia is inflammation of the lower part of the spinal cord. I read yesterday in a medical journal an account of a man who had pain in the lower part of the back, and in the course of a fortnight he became completely paralytic in his lower limbs. On examining the body after death the spinal cord was found softened, there was blood extravasated here and there, and it was said that the spinal cord bore markes of inflammation; but I am inclined to believe that inflammation of the membranes is a more common cause of paraplegia than inflammation of the spinal cord itself.

I have known a severe attack of lumbago to be followed by an attack of paralysis. I was consulted by a gentleman who had what was called severe lumbago. I only saw him once, and that in consultation, and I recommended that he should be cupped and take mercury. Some time afterwards I was asked to see him again, and then there was entire paralysis of the lower limbs. He remained in that state for some years, and then he died. After seeing this gentleman a second time, and whose case was clearly one in which severe lumbago was followed by paraplegia, I went to the house of the late Dr. Davies, of the London Hospital, to see his preparations, and amongst them was one of spinal marrow with the membranes, the lower part, especially about the cauda equina, being encrusted with coagulated lymph. On making inquiry about the preparation he said that it was rather a curious case—that the patient had had violent pain in the loins, which was followed by paraplegia—that he died, and those were the morbid appearances. In fact, he described exactly the case of the paraplegic gentleman whom I had just visited. I have seen, I will not say several, but some cases of severe lumbago in which the patient was threatened with paraplegia, but recovered under the employment of proper treatment. There was a gentleman who had some rheumatic complaint for which he used a liniment made of tincture of cantharides. One day, by mistake, he swallowed a bottle of liniment instead of his medicine. He soon found that he had got something monstrously hot in his stomach. He had to obtain advice, and then an emetic had to be procured, so that three-quarters of an hour were lost, and by that time the tincture of cantharides had nearly passed out of the stomach. Immediately afterwards he was seized with pain in the loins, there was strangury, great pain and difficulty in making water, and this was followed by partial paraplegia; by making an effort he could walk about. I conclude that the operation of the cantharides produced inflammation of the lower part of the spinal cord. Whether he recovered or not I do not know.

There is no doubt that paraplegia sometimes occurs as the result of functional disease. For example, a young lady, very delicate, with nervous symptoms, weak bodily powers, and an hysterical constitution, and whose sister laboured under an hysterical affection of one limb, began to be weak in her lower limbs, and walked about with some difficulty. The pulse became very small, her hands and feet cold, her appetite bad: she was one of those young women with whom we so commonly meet in the affluent classes of society, and sometimes in the lower. Finding this difficulty in walking about, and being little disposed to it, much more inclined to lie on the sofa, ready to avail herself of an excuse for not making exertion, she consulted a physician in the country, who told her that she had better use crutches. Her limbs then became paralytic, so that she could not stand, and it was supposed that there was disease in the spine. I went to see her, and after taking great pains I concluded that it was one of those cases so common among hysterical women. I advised that her attention should be called to her case as litle as possible, that she should take steel from time to time, that she should be encouraged to use her limbs, that the crutches should be taken away, and a bar put across the room, by holding which she might walk along, and under this treatment she, in the course of a considerable time, walked about. She continued delicate, but the paralytic symptoms were gone. A poor girl was in this hospital, under the care of Dr. Seymour, for what he considered a mere hysterical and nervous affection of the limbs—a girl that wanted tonics, steel, and good diet. She went out of the hospital; some person under whose care she came, thought that paralysis was coming on, and he cupped her again and again, blistered her, and kept her low. All the time that this treatment was pursued she got worse, and she came into the hospital again with her lower limbs paralytic, with large sloughs on the nates and ankles, and she died. On examining the body after death we could find no morbid appearances whatever, and, taking the history of the case and the post mortem examination together, I cannot but believe that the disease under which she laboured was that general want of nervous energy to which hysterical young women are liable, and that the aggravation of the symptoms was the consequence of injudicious treatment by taking away blood from a person who rather wanted blood put into her, and by tormenting her with other painful remedies.

These are the principal causes of paraplegia affecting the lower limbs, so far as I have had an opportunity of observing the disease. I need not tell you that diseases in the vertebræ will produce paraplegic symptoms; but it is not my intention at this moment to enter on diseases of the spine.—*London Lancet.*

